# Seeds of *Stevia rebaudiana* Bertoni as a Source of Plant Growth-Promoting Endophytic Bacteria with the Potential to Synthesize Rebaudioside A

**DOI:** 10.3390/ijms24032174

**Published:** 2023-01-21

**Authors:** Magdalena Simlat, Agata Ptak, Anita Jaglarz, Agnieszka Szewczyk, Michał Dziurka, Artur Gurgul

**Affiliations:** 1 Department of Plant Breeding, Physiology and Seed Science, University of Agriculture in Krakow, Łobzowska 24, 31-140 Krakow, Poland; 2 Moredun Research Institute, Pentland Science Park, Bush Loan, Penicuik EH26 0PZ, UK; 3 Department of Pharmaceutical Botany, Faculty of Pharmacy, Jagiellonian University Medical College, Medyczna 9, 30-688 Krakow, Poland; 4 The Franciszek Górski Institute of Plant Physiology, Polish Academy of Sciences, Niezapominajek 21, 30-239 Krakow, Poland; 5 Centre for Experimental and Innovative Medicine, University of Agriculture in Krakow, Rędzina 1c, 30-248 Krakow, Poland

**Keywords:** genome, growth regulators, endophytic bacteria, rebaudioside A, seeds, stevia

## Abstract

In this study, a new strain of *Pantoea vagans*, SRS89, was isolated from surface-sterilized stevia seeds. The isolate was evaluated using morphological, molecular, and biochemical methods. The bacterium was 1.5 μm long, yellowish in color, and classified as Gram-negative. Whole genome sequencing of our strain revealed the presence of a 4,610,019 bp chromosome, and genome annotation resulted in the detection of 4283 genes encoding 4204 putative coding sequences. Phylogenic analysis classified the genome of our strain close to the MP7 and LMG 24199 strains of *P. vagans*. Functional analysis showed that the highest number of genes within the analyzed bacterium genome were involved in transcription, amino acid transport and metabolism, and carbohydrate transport and metabolism. We also identified genes for enzymes involved in the biosynthesis of carotenoids and terpenoids. Furthermore, we showed the presence of growth regulators, with the highest amount noted for gibberellic acid A_3_, indole-3-acetic acid, and benzoic acid. However, the most promising property of this strain is its ability to synthesize rebaudioside A; the estimated amount quantified using reversed-phase (RP)-HPLC was 4.39 mg/g of the dry weight of the bacteria culture. The isolated endophytic bacterium may be an interesting new approach to the production of this valuable metabolite.

## 1. Introduction

*Stevia rebaudiana* Bertoni (Asteraceae) is an important plant due to its content of steviol glycosides (SGs), a kind of non-caloric and intensely sweet chemical compounds that were approved for human consumption by the Food and Drug Administration (US) in 2008 and by the European Union in 2011. The SG biosynthetic pathway in stevia has already been described [1,2,3]. The natural properties of SGs make them the subject of increasing interest as a natural sweetener whose consumption could exert beneficial effects on human health [4,5,6]. In stevia, more than 30 SGs have been identified, with stevioside (ST) and rebaudioside A (Reb A) being the most significant [7,8,9,10,11,12]. As reported by DuBois and Stephenson, the taste quality of Reb A is better than that of ST because it is sweeter and less bitter [13]. Reb A can be used in various foods or dietary supplements as a sweetener. In addition, studies have shown that Reb A attenuates aging by acting as an effective cellular antioxidant and lowering the ectopic accumulation of neutral lipids [14]. Recently, Reb A has also been demonstrated to have many physiological functions, such as antihypertension, anti-diabetes, and anticaries [15]. Research is also being conducted on the effect of Reb A and other SGs on intestinal microbiota [16,17]. The SGs are found in the leaves, where they are detected in the highest numbers and amounts, as well as in other organs with declined order: flowers, stems, seeds, and roots [18]. The concentrations of SGs in stevia leaves vary based on genotype, plant growth stage, fertilization level, and growing condition [19,20]. Stevia is naturally propagated by seeds; however, poor germination and the long time required for the development of seedlings suitable for planting in the field (which can last up to 60 days) means that it is not a widespread method in commercial stevia production. The possibility of improving stevia seed germination using physical factors or plant growth regulators has already been published [21,22,23,24,25,26]. As reported by Puente et al. [27], endophytic bacteria might also increase the rate of seed germination.

Endophytic bacteria are defined as organisms that live most of their lives inside plant tissues without eliciting any pathogenic symptoms [28]. As reported by Smith et al. [29], among the over 300,000 plant species found worldwide, each contains endophytes. Endophytic bacteria have been isolated from the leaves, stems, roots, flowers, fruits, and seeds of various plant species [30,31,32,33]. In light of this information, it is not surprising that endophytic bacteria have also been identified in stevia plants [34,35]. As reported by Yu et al. [36], in the seedling stage, the dominant genera of the endophytic bacteria of stevia are *Enterobacterium* and *Erwinia*, while *Methylobacterium* and *Sphingomonas* were found to be the principal endophytes in mature leaves. Interestingly, its concentration was positively correlated with stevioside content and with *UGT74G1* and *UGT76G1* gene expression [36]. These genes encode UDP-glycosyltransferases, which are engaged in the last stages of the SGs biosynthesis pathway. Additionally, in the research of Oviedo-Pereira et al. [35], reinoculation with the endophytic bacteria *Enterobacter hormaechei* increased SG synthesis, as well as flavonoid accumulation in the trichomes of *S. rebaudiana* plants. However, there is no information about the endophytic bacteria that occupy stevia seeds. Endophytic bacteria were isolated from the seeds and seedlings of eucalyptus species and hybrids [27,37]. In maize seeds, Gamma proteobacteria represent the most abundant class of endophytes, most of which are *Pantoea* and *Enterobacter* [38,39]. In rice seeds, one of the dominant genera of endophytic bacteria was also found to be *Pantoea* [40]. Scientists have suggested that endophytic bacteria from seeds are transferred vertically during subsequent germination inside the plant and further affect plant growth [41,42]. Various studies have shown that endophytes can regulate plant growth with nitrogen fixation, phosphate solubilization, siderophore, 1-aminocyclopropane-1-carboxylate (ACC) deaminase activity, and indole-3-acetic acid (IAA) synthesis [43,44,45,46]. It is also well known that endophytic bacteria can produce plant growth regulators and the same or similar secondary metabolites as their hosts [47,48]. Endophytic bacteria may also enhance plant tolerance against abiotic stresses, such as salinity, drought, cold, or heavy metal toxicity [49]. However, the interactions between plants and bacteria are often specific, and the endophytic bacteria of one plant species may be pathogenic to other plant species [50].

The aim of this study was to identify and characterize endophytic bacteria isolated from surface-sterilized seeds of *Stevia rebaudiana* Bertoni to elucidate their possible beneficial role in the host plant and to potentially use them as a source of valuable metabolites. To our knowledge, this is the first mention of endophytic bacteria from stevia seeds.

## 2. Results

### 2.1. Preliminary Identification of Endophytic Bacteria

**Figure 1:**
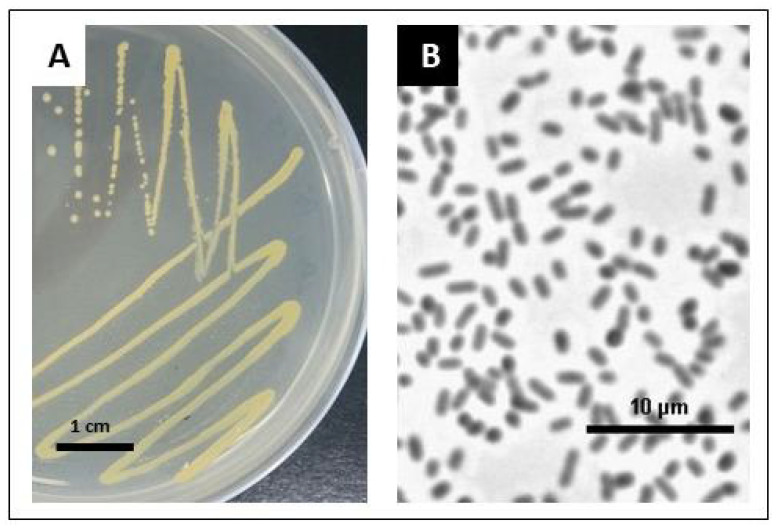
Bacteria cultures grown overnight on the LB medium at 25 °C for single colony isolation. Bacteria cultures grown overnight on the LB solid medium (**A**). Single bacteria cells after 24 h of growth in the LB liquid medium (phase contrast microscopy was performed using a Nikon Eclipse E800 microscope at 40× magnification) (**B**).

The bacteria isolated from surface-sterilized stevia seeds have rod-shaped cells about 1.5 μm long and yellowish in color (Figure 1). Bacteria were classified as Gram-negative and were further identified via sequence analysis of the PCR product obtained with universal primers for the 16S rRNA coding gene. The basic local alignment search tool for nucleotides (BLASTn) search score analysis suggested that the bacteria strain isolated from stevia seeds belongs to the genus *Pantoea*. Our strain showed the greatest similarity to the strains of *P. agglomerans* and *P. vagans* (Table S1, Figure S1 in Supplementary File S1). We named the isolated strain SRS89.

### 2.2. New Strain SRS89 of Pantoea Vagans—Genome Features, Comparative Genome Analysis, and Gene Classification

Genomic DNA of the isolated strain was sequenced, and filtered reads were assembled into 158 contigs, 143 of which were longer than 1000 bp. The N50 for the assembly was 57,973, and the L50 was 23. The total assembly length was 4,610,019 bp, with an average GC content of 55.26% (Table 1). The average assembly coverage by the reads was 22.4×. Genome annotation with NCBI Prokaryotic Genome Annotation Pipeline (PGAP) resulted in the detection of 4283 genes encoding 4204 putative coding sequences (CDS) (NCBI WGS Project JAAALG01). Furthermore, 67 tRNA-coding genes, five rRNA coding genes, and one tmRNA coding gene were identified in the analyzed sequences. Analysis with Operon-mapper software resulted in the detection of 2312 operons (Supplementary File S2), and the genes belonged to 2272 unique clusters of orthologous groups of proteins (COGs).

**Figure 2:**
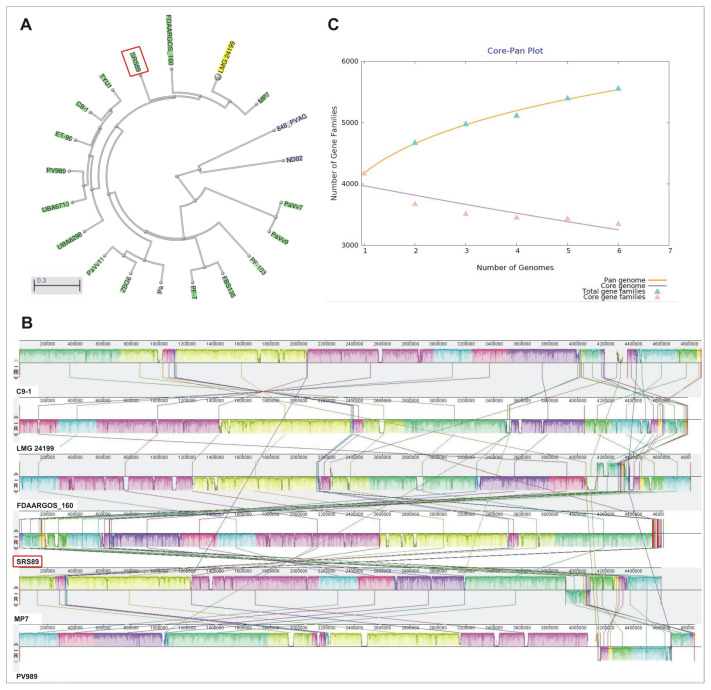
Dendrogram created based on genomic BLAST analysis for all *Pantoea vagans* strains currently (December 2022) present in the NCBI database (**A**). Genome alignment of selected *P. vagans* strains using Mauve software (http://darlinglab.org/mauve/user-guide/viewer.html, accessed on 10 January 2023) (**B**). Pan-/Core-genome plot for the six compared *P. vagans* strains (**C**).

To phylogenetically classify the isolated strain and find the best reference genomes for comparative analysis, a genomic BLAST-based dendrogram was used. This analysis allowed for the selection of closely related *P. vagans* strains for comparative analysis. The strains included FDAARGOS_160, LMG24199, MP7, C9-1, and PV989 (Figure 2A). The visualization of our SRS89 strain genome with respect to the C9-1 strain revealed not only several highly homologous blocks but also the inversion of a genomic segment and several smaller rearrangements. Some of the other compared strain genomes showed more complex rearrangements (Figure 2B). Among the compared strains, SRS89 seemed to have a rather lower genome size (4.6 Mb), with a smaller genome observed only for the MP7 (4.59 Mb) strain (Table 2). The GC content of the analyzed genomes was comparable for all reference strains and oscillated within a range of 55.1–55.3%. The number of identified coding sequences (CDS) was the lowest in the MP7 strain (4005) and the highest in PV989 (4397), with an intermediate value for SRS89 (4204). The whole pan-genome for the compared bacteria involved 5562 genes, of which 3352 (60.2%) constituted a common core-genome (Figure 2C). The number of accessory genes ranged from 451 in SRS89 to 619 in C9-1. The number of strain-specific genes in the compared dataset of strains ranged from 102 to 349 and was 159 for the SRS89 strain (Supplementary File S3). Additionally, as many as 85 CDS were exclusively absent in this strain (Table 2).

**Figure 3:**
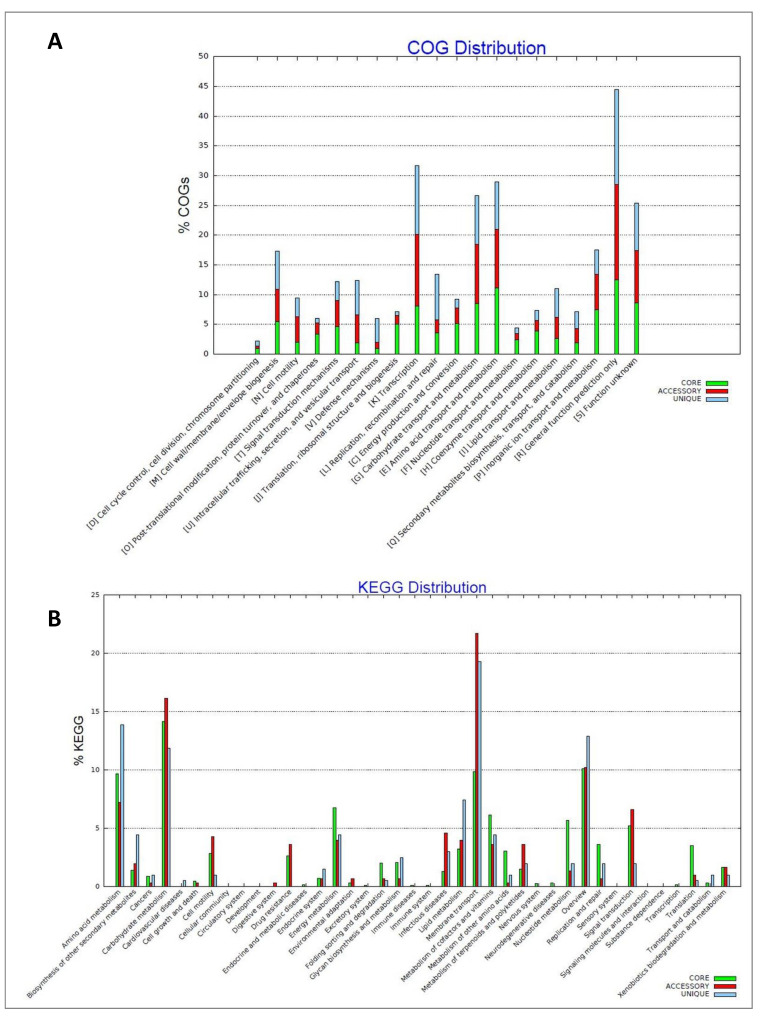
Distribution of orthologous groups of proteins (COGs) across the analyzed *Pantoea vagans* strains pan- and core-genome (**A**). Distribution KEGG pathways across the analyzed *P. vagans* strains pan- and core-genome (**B**).

Functional analysis of a pan- and core-genome showed that the highest number of genes within the analyzed genomes were engaged in transcription, amino acid transport and metabolism, and carbohydrate transport and metabolism (Figure 3A), with a significant number of genes related to COG involved in membrane transport (Figure 3B).

Regarding some details, in the SRS89 strain we detected genes involved in the early steps of the 2-C-methyl-D-erythritol-4 phosphate (MEP) pathway that is used, among others, for terpenoid biosynthesis. In stevia plants, that pathway is also used for SG biosynthesis. The detected genes were: *dxs* coding for geranylgeranyl diphosphate synthase type II [EC: 2.5.1.29] and 1-deoxy-D-xylulose-5-phosphate synthase [EC:2.2.1.7], *dxr* coding for 1-deoxy-D-xylulose-5-phosphate reductoisomerase [EC:1.1.1.267], and *idi* coding for isopentenyl-diphosphate delta-isomerase [EC:5.3.3.2]. The genes engaged in carotenoid biosynthesis were also identified: *zep* coding for zeaxanthin epoxidase [EC:1.14.13.90], *crtB* coding for phytoene synthase [EC:2.5.1.32] and *crtZ* coding for beta-carotene 3-hydroxylase [EC:1.14.13.129] (Supplementary File S4).

**Figure 4:**
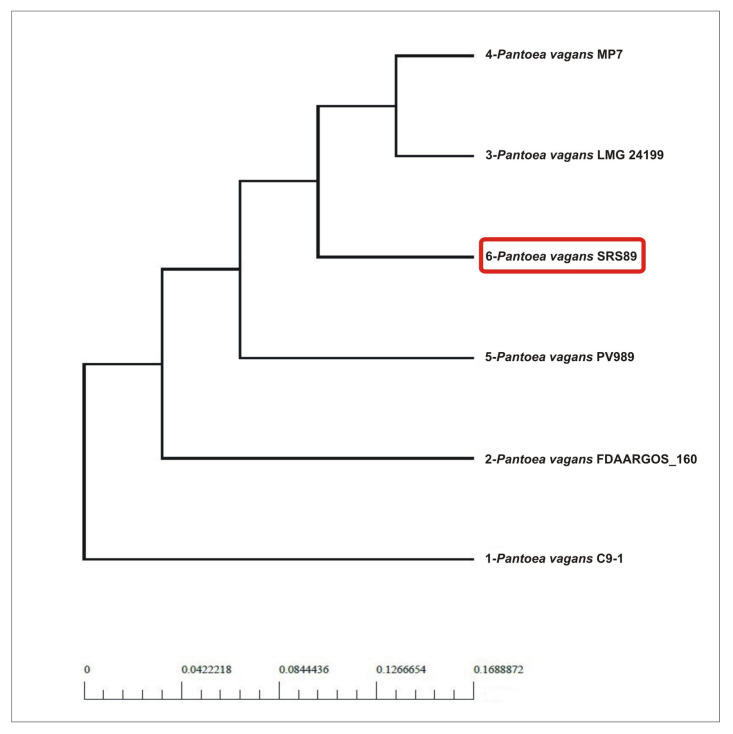
Phylogenetic analysis the selected *Pantoea vagans* strains based on pan- and core-genome analysis.

The phylogenic relationships among the compared strains were additionally visualized via pan- and core-genome analyses. Similarly, as in the initial phylogeny analysis results, the pan-genome analysis clustered SRS89 in a separate clade at a long distance to C9-1, while the core-genome-based analysis clustered SRS89 as genetically more like MP7 and LMG24199 (Figure 4). A relatively large pan-genome-based distance was observed between SRS89 and FDAARGOS_160, which were clustered in a direct vicinity in genome-based analysis.

### 2.3. Biochemical Properties of SRS89 Strain

**Figure 5:**
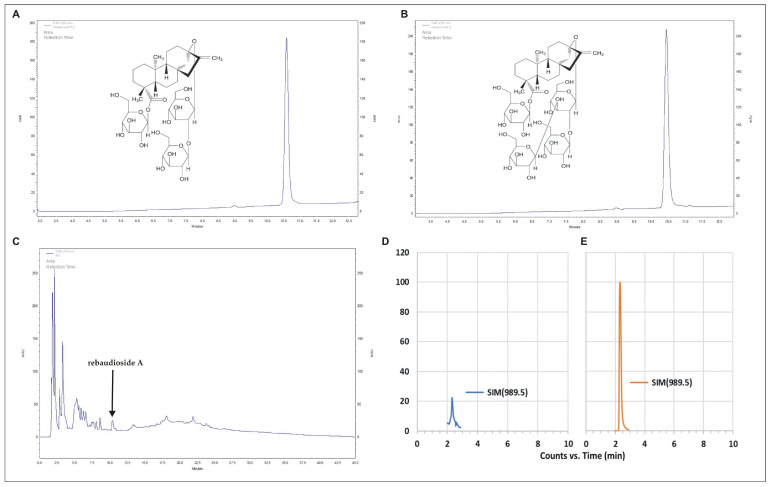
RP-HPLC chromatograms of the stevioside (**A**), rebaudioside A (**B**) standards, and steviol glycosides fraction extract from the SRS89 strain of *Pantoea vagans* (**C**). Single ion [M+Na]^+^ monitoring (SIM) chromatogram of bacteria sample (**D**) and pure rebaudioside A standard (**E**) separated in HILIC mode.

The reversed-phase (RP)-HPLC analyses showed that the strain SRS89 of *P. vagans* contains Reb A. It was the only SG we identified in this bacterium, and its estimated amount quantified using RP-HPLC was 4.39 mg/g (±0.2) of the dry weight (DW) of the bacteria culture. We confirmed the presence of Reb A using Electrospray Ionization Mass Spectrometry (EMI-MS) analysis (Figure 5). Additionally, by using the RP-HPLC analyses, we demonstrated the presence of phenylalanine and tryptophan at concentrations of 0.75 mg/g DW (± 0.2) and 0.57 mg/g DW (±0.07), respectively (Table 3).

**Figure 6:**
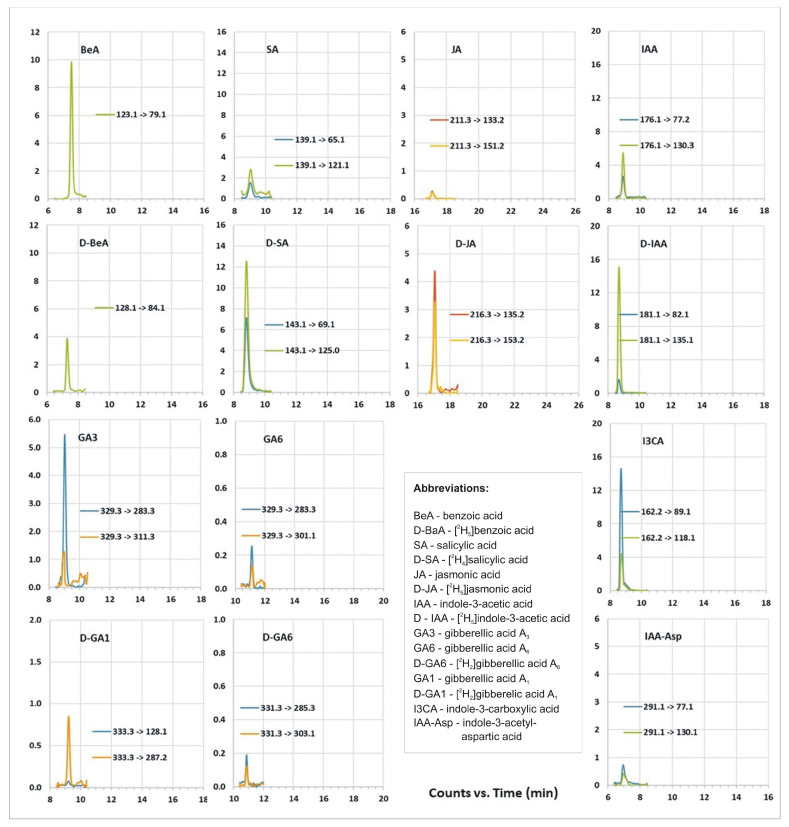
UHPLC-MS/MS growth regulator profile of bacterial extract, chromatograms of multiple reaction monitoring (MRM) transitions for the analyzed plant growth regulators and related substances.

Ultrahigh-performance liquid chromatography–tandem mass spectrometry (UHPLC-MS/MS) analyses enabled us to identify growth regulators in bacteria extract such as indole-3-acetic acid (IAA, 831.7 pg/mg DW), indole-3-carboxylic acid (I3CA, 3716.6 pg/mg DW), indole-3-acetyl-aspartic acid (IAAsp, 112 pg/mg DW), gibberellic acid A_3_ (GA_3_, 17,402 pg/mg DW), gibberellic acid A_6_ (GA_6_, 318 pg/mg DW), benzoic acid (BeA, 10,770 pg/mg DW), salicylic acid (SA, 1830 pg/mg DW), and jasmonic acid (JA, 213 pg/mg DW) (Figure 6, Table 3). We were also able to detect trace amounts of abscisic acid (ABA) and auxins such as indole-3-acetyl-glutamic acid (IAGlu), indole-3-acetonitrile (IAN), indole-3-propionic acid (IPA), and some gibberellins: GA_1_, GA_4_, GA_19_, GA_44_, and GA_53_ (Table 3).

### 2.4. Seed Germination Promoting Potential of SRS89 Strain

**Figure 7:**
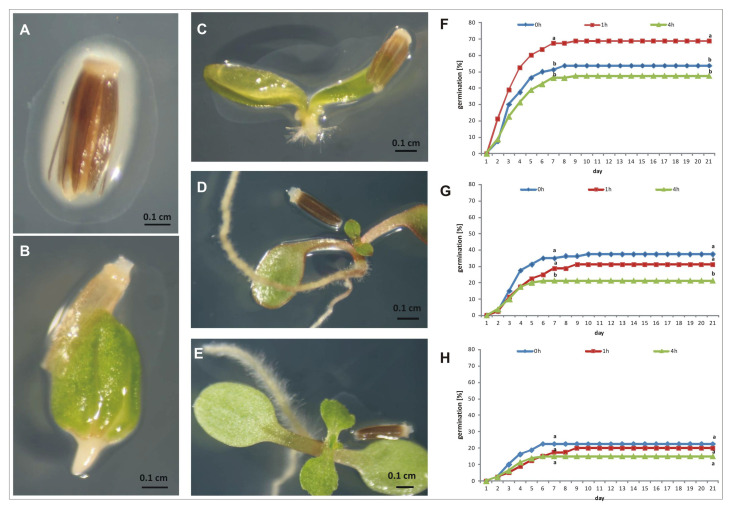
The effect of SRS89 strain on stevia seed germination. Seeds were surface sterilized and inoculated with the isolated *Pantoea vagans* strain for one or four hours. Non inoculated seeds were used as a control. The following stages of stevia seed germination after inoculation with SRS89 strain (**A**–**E**). The stevia seed germination on agar gel (**F**), agar gel supplemented with 50 mM NaCl (**G**) or with 150 mM NaCl (**H**). The values of seed germination are means of four replications (*n* = 4). Different letters indicate a significant difference in the percentage of germinated seeds at the 7th (germination energy, GE) and 21st (germination capacity, GC) day of incubation at *p* < 0.05 according to Duncan’s test.

The stevia seeds germinated properly after reinoculation and produced a well-developed root system with a large number of root hairs (Figure 7A–E). It can be seen that one hour of inoculation significantly promoted seed germination. It was visible from the second day of the experiment that the maximum value of germinated seeds reached 67.5% on the 9th day of germination compared to 51% of the non-reinoculated seeds. However, extending the inoculation time to 4 h decreased germination (Figure 7F). When the seeds were germinated on agar gel (AG) containing 50 mM NaCl, one-hour inoculation has no effect on germination when compared with the control, but four-hour inoculation significantly decreased the germination capacity (GC) (Figure 7G). In the experiment in which AG containing 150 mM NaCl was used, we observed a significant reduction in seed germination when compared to seed germination on other substrates. Here, however, the effect of inoculation on seed germination was not visible, and the GC was, on average, 19% (Figure 7H). We also observed that NaCl added to AG had a negative effect on the further growth of the seedlings, which was particularly evident when the concentration of NaCl was 150 mM. Here, the seedlings were small, poor in quality, and died shortly after germination.

## 3. Discussion

Endophytic bacteria inhabit plant tissues without causing any harm to the host [51]. Numerous studies have revealed that these bacteria play a crucial role in the growth and development of a wide variety of host plant species. They also increase plant tolerance for environmental stresses and inhibit plant pathogen growth. These roles are mainly performed by secreting growth regulators and consequently helping to improve nutrition. Some previous papers have described the isolation of endophytic bacteria from different organs of stevia plants [34,35,36]; however, until now there has been no information about the endophytic bacteria inhabiting the stevia seeds. Here, for the first time, we report on bacteria isolated from *Stevia rebaudiana* seeds. Based on a sequence homology analysis of the 16S rRNA coding gene, we have shown that our bacterium shows the highest similarity to *P. agglomerans* and *P. vagans*. The verification carried out with the use of whole genome sequencing (WGS) confirmed that is definitely *P. vagas* strain. The genus *Pantoea* is a member of *Enterobacteriales* and represents several species that have been isolated from numerous different sources, mostly plants [52]. Anjum and Chandra [34] have already reported isolation from stevia plants of the bacteria belonging to the genus *Pantoea*, although species identification was not performed in that work. Originally, *Pantoea* was known as a plant pathogen; however, today, it is generally known that these bacteria have a beneficial effect on plants, especially in growth promotion and phytopathogen control [53,54,55,56]. *P. vagans* is very often isolated together with *P. agglomerans* since both species occupy the same ecological niches, and even some isolated *P. vagans* strains were previously identified as *P. agglomerans* [57]. As reported by Palmer et al. [58], *P. vagans,* in contrast to some strains of *P. agglomerans,* is not able to induce galls and is not a pathogenic bacterium. The bacteria belonging to the genus *Pantoea* were identified as the major endophytes of maize [39,59] and wheat [54] seeds. Additionally, *P. agglomerans* was isolated in the seeds of switchgrass [60]. One of the best-known *P. vagans* strains used in practice is the C9-1 strain isolated from apples (*Malus x domestica* ‘Jonathan’) (Michigan). This strain is the main component of the registered preparation BlightBan C9-1 (Nufarms America Inc., Burr Ridge, IL) for the biocontrol of fire blight caused by the related enterobacterium *Erwinia amylovora* [61]. As reported by Smits et al. [61], the *P. vagans* C9-1 genome does not contain known virulence determinants of enterobacteria, such as toxins, protolytic enzymes, or type II secretion system effectors. The strain of *P. vagans* isolated in our research—SRS89—was able to synthesize several types of growth regulators with great importance for plant growth, among which GA_3_ was the most abundant. Previous research has reported different *Pantoea ssp.* are able to produce growth regulators such as GA_3_ and ABA [62], IAA [54], cytokinins [63], ACC [64], and to phosphate solubilization or nitrogen fixation [54,65]. GAs have been recognized as regulators in numerous aspects of plant growth and development. The first reported bacterial strain with the ability to produce GA was *Rhizobium phaseoli* [66]. However, several other species with ability to biosynthesis active GAs have also been identified [67,68,69,70,71,72]. Here, for the first time, we indicated that strain SRS89 of *P. vagans* isolated from stevia seeds can synthesis GA_3_ and GA_6_. It has also been determined that the GA biosynthesis pathway in bacteria is identical to the 13-hydroxylation pathway found in plants, although the presence of ferredoxine (Fd) and short-chain alcohol dehydrogenase reductase (SDR) genes is specific to bacteria [73]. However, in plants, fungi, and bacteria, the biochemical route for GA synthesis starts from geranyl-geranyl diphosphate (GGPP) via isopentenyl-diphosphate (IPP), which is a building block for all terpenoid/isoprenoid compounds [74]. IPP can be generated via the MEP pathway, which in stevia plants is also used for SG biosynthesis [2]. We also detected some genes involved in the early steps of that pathway (*dxs*, *dxr,* and *idi*). It is also interesting that strain SRS89 isolated from stevia seeds seems to be able to perform Reb A biosynthesis. This is the first information about the capability of endophytic bacteria to produce a kind of SG. In the last few years, there have been reports of the possibility of synthesizing SGs by bacteria or fungi; however, this ability was obtained by genetic transformation of model species of microorganisms [75,76,77,78]. Therefore, further research is needed to understand the biosynthetic pathway of these compounds in the *P. vagans* SRS89 strain. According to all forecasts, the demand for SGs will increase, not only due to the projected increase in the number of diabetic patients but also due to changes in food preferences. However, in many countries, due to climatic conditions, the cultivation of stevia is either impossible or unprofitable. The use of endophytic bacteria for the production of SGs brings many benefits, including low cost, easily scaled-up production, shortening the time, and synthesis independent of environmental conditions. Recent studies have demonstrated that endophytes may be a rich source of natural products for medicinal, industrial, and agricultural use [79,80,81,82,83,84]. Numerous bioactive compounds produced by endophytes have already been commercialized and have been found useful in agriculture and pharmacology [85,86,87,88].

In the SRS89 strain of *P. vagans,* we also detected IAA and indole-derivative metabolite indole-3-carboxylic acid (I3CA). IAA is the most common auxin in plants and plays a key role in regulating numerous processes related to growth and development. IAA elicits seed growth by cell elongation. Depending on the type of organ and the developmental stage of the plant, IAA can be synthesized either independently or dependently of tryptophan. We also detected tryptophan in our bacteria strain, so we can conclude that it may be used for IAA synthesis. It should be noted that tryptophan is not detected in stevia plants [89]. It is worth mentioning that most auxins occur in plants as auxin conjugates. One of the major auxins, IAA, is inactivated to IAA-aspartate (IAAsp) via ATP-dependent conjugation catalyzed by amidosyntethases from the Gretchen Hagen 3 (GH3) acyl-adenylating enzyme family. Bacteria also have the ability to produce auxin. This ability concerns mainly plant growth-promoting rhizobacteria (PGPR) and endophytes. Our study showed that IAA production is also a feature of SRS89 strain isolated from stevia seeds. The ability to produce IAA was previously detected in different bacteria isolated from the rhizosphere of *Stevia rebaudiana* [90]. Some researchers have suggested that plants are colonized by high numbers of IAA-producing bacteria [40,49,91]. Some reports have also shown that inoculation of IAA-producing endophytic bacteria is a promising way to enhance plant biomass, root length, root tip number, and root surface area [47,92].

The other growth regulator that we identified in the SRS89 bacteria strain was jasmonic acid. Jasmonates (JAs) are lipid-derived signaling molecules produced by certain bacteria, fungi, and plants. Plants synthesize JAs in response to developmental cues or environmental stress. They take part in the regulation of many physiological processes, including seed germination, root growth, organ formation, flowering, fruit ripening, and leaf aging [93]. While much information exists on the biosynthesis and function of JA in plants, such knowledge regarding microorganisms is still scarce [94].

We also analyzed the effect of the identified strain on stevia seed germination. It is known that some endophytic bacteria have potential as biostimulators of crop species [40,49,95,96]. The surface-sterilized *S. rebaudiana* seeds were reinoculated with a bacterial culture before germination. Stevia belongs to a salt-sensitive species [26,97], and some evidence exists that endophytic bacteria promote plant growth in salt-stress conditions [98]. These beneficial functions are mainly performed by supplying nutrients, the detoxification of harmful compounds, and the production of bioactive compounds, such as secondary metabolites and hormones. The beneficial effect of bacteria on germination was observed only for control conditions. When there was no NaCl in the germination medium, inoculated stevia seeds germinated 15% better than non-reinoculated seeds. However, when NaCl was present in the medium, we did not observe a beneficial effect. This may indicate that the bacterium is sensitive to salinity and its lack of activity results in lower germination of stevia seeds.

## 4. Materials and Methods

### 4.1. Bacteria Isolation and Maintenance

The stevia seeds (POLAN Breeding and Seed Company, Krakow, Poland) were surface sterilized [23] and then placed in Petri dishes (7 cm in diameter) containing sterile solidified lysogeny broth (LB) medium (Sigma-Aldrich, Saint Louis, MO, USA). To confirm that the sterilization process was successful, 100 μL of the water used for the final washing of the surface-sterilized seeds was placed on the same medium and examined for microbial growth during incubation (25 °C for seven days). After sterilization, the seeds were incubated in sterile conditions at 25 °C for seven days. During this time, yellow bacteria appeared around some of the seeds. Bacterial colonies were picked up and further purified by repeated streaking on the same medium. A single bacterial colony was isolated and used to inoculate 5 mL of Tryptic Soy Broth culture medium (Oxoid, Basingstoke, UK). After 24 h of incubation, 4 mL of the bacteria culture was centrifuged at 4 °C with 38,903× *g*, for 15 min (Sigma 3–16 K), and the obtained pellet was used for genomic DNA extraction. To determine the DNA extraction method, 100 µL of bacteria culture was grown overnight on an LB solid medium at 25 °C. A single bacteria colony was isolated and stained using the standard Gram technique for microscopic observation. The remaining volume of bacterial culture was transferred to new tubes and stored at −80 °C in 20% (*v*/*v*) glycerol. DNA was extracted using the DNeasy Blood and Tissue Kit (Qiagen, Hilden, Germany) according to the manufacturer’s protocol. Bacteria DNA concentration was assessed spectrophotometrically using NanoDrop One (Thermo Scientific, Waltham, MA, USA).

For bacteria identification, preliminary analysis using the 16S rRNA gene sequence similarity was performed, followed the detailed characteristics based on the whole genome sequence (WGS) data. Biochemical analysis of the isolated strain was also carried out and the ability to increase the germination capacity of stevia seeds was assessed.

### 4.2. Amplification and Sequencing of the 16S rRNA Gene

Bacteria species identification was performed according to Marchesi et al. [99]. For the amplification of the 16S rRNA gene, the specific par of primers 63F (5′-CAGGCCTAA CACATGCAAGTC-3′) and 1387R (5′-GGGCGGWGTGTACAAGGC-3′), was used. PCR was performed in 50 µL of a reaction mixture containing 1× GoTaq® Green Master mix, 1 µM of each primer, and <250 ng of bacteria genomic DNA. PCR was conducted as follows: Five min of initial denaturation at 95 °C followed by 30 cycles of denaturation for one min at 95 °C, annealing for one min at 55 °C, and elongation for 1.5 min at 72 °C, followed by a final five-min extension step at 72 °C (Biometra Tone; Analytik Jena, Jena, Germany). The PCR product was visualized via 1% agarose gel electrophoresis with GelRed (Biotium, Fremont, CA, USA) for DNA staining. The PCR product was excised from the gel and cleaned with a QIAquick PCR purification kit (Qiagen, Hilden, Germany). Its concentration was assessed spectrophotometrically (NanoDrop One; Thermo Scientific, Waltham, MA, USA) and adjusted to 10 ng/μL prior to sequencing (MWG, Birmingham, UK). The obtained sequence was analyzed with BLASTn using a database called “16S ribosomal RNA sequences (Bacteria and Archaea)” (NCBI, http://blast.ncbi.nlm.nih.gov/Blast.cgi (accessed on 3 January 2020)).

### 4.3. WGS Isolated Bacteria Strain and Data Analysis

Genomic DNA obtained from the endophytic bacteria was used for WGS. Sequence libraries were prepared using the Nextera XT v2 library preparation kit and sequenced on a MiSeq desktop sequencer (Illumina, San Diego, CA, USA) using MiSeq V3 reagent kits. The library was sequenced in a 300 bp paired-end run to obtain 404,752 reads. Raw reads were processed using Trimmomatic software [100] to remove adapter sequences (only keeping reads longer than 30 bp after timing) and filtered for quality to remove reads of a quality lower than Q20. Additionally, only reads with pairs after filtering were retained for further analysis. This resulted in 344,414 300-bp reads that were used for genome assembly. The initial assembly was created using Unicycler [101] software and the SPAdes algorithm [102] with an error correction step, in Pilon [103] for the polishing of the final assembly. Subsequently, Quast software [104] was used to evaluate assembly quality. The resulting contig annotation was done using NCBI PGAP. Additional annotation and comparative analysis were performed using the RAST server [105]. Operons were identified using the Operon-mapper software [106]. Taxonomic analysis of the studied strain was performed using a dendrogram created based on genomic BLAST available in the NCBI database in the *Pantoea vagans* genome data Table Using this approach, we identified closely related *Pantoea vagans* strains—FDAARGOS_160 (GCA_001558735.2), LMG24199 (GCA_004792415.1), MP7 (GCA_000757435.2), and slightly more distant C9-1 (GCA_000148935.1) and PV989 (GCA_003032455.1)—which were further used for comparative analysis. For visualization purposes using Mauve software [107] and for genome submission, the contigs of the analyzed strains were scaffolded against the C9-1 strain assembly (GCA_000148935.1) using MEDUSA software [108]. Pan- and core-genome analyses, as well as pan- and core-phylogeny, were performed for all the selected stains specified above using BPGA software [109]. The obtained contigs were deposited in the GenBank database under the assembly accession numbers: GCA_009905795.1, ASM990579v1, and the strain was labeled as SRS89.

### 4.4. HPLC Analysis of Isolated Bacteria

To prepare the bacteria for HPLC analysis, 100 µL of bacteria culture stored at −80 °C was used. Bacteria were grown overnight on an LB solid medium at 25 °C and then streaked onto an LB solid medium for overnight culturing at 25 °C. Single colonies were used to inoculate 100 mL of liquid LB medium and cultured for the next 24 h at 25 °C. The obtained bacterial cultures were centrifuged at 6000× *g*, and the resulting pellet was stored at −80 °C before lyophilization. To determine SGs and amino acids (phenylalanine and tryptophan) using RP-HPLC analysis, 250 mg of dry biomass was extracted according to Simlat et al. [26]. The analyses were performed in three replications.

### 4.5. Mass Spectrometry Confirmation of RebA

Samples in methanol were evaporated to dryness under N_2_ and redissolved in acetonitrile/water (3/1 *v*/*v*). Separation was achieved in hydrophilic interaction liquid chromatography (HILIC) mode on BlueOrchid NH2 (100 × 2 mm, 1.8 µm, Knauer, Berlin, Germany) in gradient mode of (A) 2 mM ammonium formate in water and (B) 5% 2 mM ammonium formate in acetonitrile, 0–4 min 100–75% B, 4–5 min 75–35% B, then returned to 100% B in 1 min. The system consisted of UHPLC (Agilent Infinity 1260, Agilent Technologies, Santa Clara, CA, USA) and a triple quadrupole mass spectrometer (Agilent 6410, Agilent Technologies, Santa Clara, CA, USA) with electrospray ionization (ESI). The measurements were performed in the positive mode in single ion monitoring (SIM), and the [M+Na]^+^ ion was monitored.

### 4.6. Profiling of Plant Hormones

Ultrahigh-performance liquid chromatography coupled with tandem mass spectrometry (UHPLC-MS/MS) was used for the analysis of phytohormones, according to Hura et al. [110] and Dziurka et al. [111]. A stable isotope-labeled internal standard mixture was added to lyophilized bacteria samples of about 20 mg. Samples were extracted (methanol/H_2_O/formic acid, 15:4:1 (*v*/*v*/*v*)) and then evaporated under an N_2_ stream (TurboVap LV, Caliper, Hopkinton, MA, USA). After dissolution in 3% (*v*/*v*) methanol in 1 M HCOOH, samples were cleaned up on a hybrid solid-phase extraction (SPE; Bond Elut Plexa PCX; Agilent Technologies, Santa Clara, CA, USA) column. Targeted profiling of phytohormones and related compounds was conducted in multiple reaction monitoring (MRM) mode on an Agilent Infinity 1260 UHPLC system (Agilent Technologies), coupled with 6410 QQQ LC/MS with an electrospray interface (ESI) ion source (Agilent Technologies). The separation was achieved on an Ascentis Express RP-amide analytical column (2.7 μm, 2.1 mm × 150 mm; Supelco, Bellefonte, PA, USA) in a linear gradient of H_2_O vs. acetonitrile with 0.01% (*v/v*) of HCOOH. Further technical details are given in Table 4. As internal standard, [^2^H_2_]GA_1_ (D-GA_1_), [^2^H_2_]GA_6_ (D-GA_6_) [^2^H_5_]indoleacetic acid (D-IAA), [^2^H_4_]salicylic acid (D-SA), and [^2^H_5_]benzoic acid (D-BeA) (OlChemim, Olomouc, Czech Republic), and [^2^H_5_]jasmonic acid (D-JA) (CND Isotopes, Quebec, Canada) were used.

### 4.7. Effect of Endophytic Bacteria on Stevia Seed Germination

Before inoculation, the stevia seeds were surface sterilized according to Simlat et al. [23]. The efficiency of sterilization was confirmed by plating 100 µL of the final rinse water onto LB plates and incubating them for seven days at 25 °C. After disinfection, the seeds were dried on filter paper (to a moisture content of approximately 12%) and then reinoculated by dipping them into the bacterial culture. To prepare the inoculum, 100 µL of the bacteria culture stored at −80 °C was used. Bacteria culture was grown overnight on an LB solid medium at 25 °C and then streaked onto an LB solid medium for overnight culturing at 25 °C. A single colony was used to inoculate 100 mL of the liquid LB medium and cultured for 24 h at 25 °C. After incubation, the bacteria culture was adjusted to 0.5 OD using LB liquid medium and used for seed treatment. Seed inoculation lasted either one or four hours in darkness at 25 °C, during which the seeds were mixed (130 rpm) (Innova, Fredericton, NB, Canada). After inoculation, the seeds were dried and placed on Petri dishes containing AG (dH_2_O solidified with 0.7% Difco Bacto Agar), AG with 50 mM NaCl, or AG with 150 mM NaCl. Disinfected, non-reinoculated seeds were used as controls for each germination substrate. The germination conditions in the controlled plant growth chamber were as follows: 25 °C, white fluorescent light with an intensity expressed as a photosynthetic photon flux density (PPFD) of 60 μmol m^−2^s^−1^ (which we previously tested: [23]) for 12 h/day, and 70 ± 5% of relative humidity (Adaptis-A1000TC, Conviron, Winnipeg, MB, Canada). The experiment was performed in four replications, each of which consisted of 20 seeds. The number of germinated seeds was recorded each day, starting from the first day after the seeds had been placed in Petri dishes. The percentage of germinated seeds after seven days of incubation was expressed as the germination energy (GE), and after 21 days as the germination capacity (GC).

### 4.8. Statistical Analysis

The obtained data (Reb A, amino acids, growth regulators content) were reported as mean ± standard deviation (SD). To determine the significant differences between inoculation time, the results for GE and GC were analyzed using STATISTICA software (version 13.1, www.statsoft.com, accessed on 10 January 2023) by one-way ANOVA, followed by Duncan’s multiple test (*p* < 0.05).

## 5. Conclusions

In summary, the present study reports on the isolation, identification, and characteristics of endophytic bacteria from *Stevia rebaudiana* Bertoni seeds. Based on the results of phylogenetic analysis based on sequencing data, the isolated strain SRS89 is considered to represent a novel strain of *Pantoea vagans*. We detected the ability of the isolated strain to promote host seed germination, and to synthesize growth regulators, among which GA_3_ was the most present. However, in our opinion, the most promising property of isolated strain SRS89 is its ability to synthesize rebaudioside A—a kind of SGs typical of stevia plants. We also found some genes involved in SG biosynthesis. The discovery of endophytic bacteria with the capacity to synthesize Reb A fits with the increasing interest in the use of microorganisms for producing valuable metabolites with health benefits as alternatives to chemical synthesis or plant-derived metabolites. Further research on the SRS89 strain should focus on optimizing the industrial-scale production of Reb A using a fermenter. Additionally, the detailed elucidation of how Reb A is synthesized in bacterial cells is valuable and should be investigated for the possibility of synthesizing other types of rebaudioside.

## Figures and Tables

**Table 1:** Quality assessment for SRS89 strain assembly.

Statistics	
# contigs	151
# contigs (≥0 bp)	158
# contigs (≥1000 bp)	143
Largest contig	175,366
Total length	4,610,019
Total length (≥0 bp)	4,611,133
Total length (≥1000 bp)	4,606,440
N50	57,973
N75	28,681
L50	23
L75	51
GC (%)	55.26
**Mismatches**	
#N’s	0
#N’s per 100 kbp	0

**Table 2:** Genome and genes statistics for the analyzed strains of *P. vagans*.

No.	Strain	Genome Size (bp)	Number of CDS	Genomic G+C %	Pan-Genome Size	No. of Core Genes	No. of Accessory Genes	No. of Unique Genes	No. of Exclusively Absent Genes
**1**	SRS89	4,610,019	4204	55.3	5562	3352	451	159	85
**2**	C9-1	4,888,338	4354	55.1			619	205	43
**3**	MP7	4,598,703	4005	55.1			484	102	17
**4**	FDAARGOS_160	4,808,855	4335	55.2			475	349	111
**5**	LMG 24199	4,790,329	4318	55.3			594	188	8
**6**	PV989	4,839,154	4397	55.3			571	264	19

**Table 3:** Steviol glycosides, amino acids and growth regulars content in *Pantoea vagans* strain SRS89. The results are the means of three replications (*n* = 3) ±SD (nd—not detectable; ta—trace amount).

Steviol Glycosides	Content [mg/g of DW] ± SD
stevioside (ST)	nd
rebaudioside A (Reb A)	4.41 ± 0.21
**Amino acids**	**Content [mg/g of DW] ± SD**
phenylalanine	0.75 ± 0.13
tryptophan	0.57 ± 0.07
**Growth regulators**	**Content [pg/g of DW] ± SD**
indole-3-carboxylic acid (I3CA)	3716.6 ± 166.2
indole-3-acetic acid (IAA)	831.7 ± 68.4
indole-3-acetyl-aspartic acid (IAAsp)	112.0 ± 16.6
indole-3-acetamid (IAM)	ta
oxoindole-3-acetic acid (OxIAA)	ta
indole-3-acetyl-glutamic acid (IAGlu)	ta
indole-3-acetonitril (IAN)	ta
indole-3-propionic acid (IPA)	ta
GA_3_	17,402.2 ± 292.8
GA_6_	318.3 ± 78.3
GA_1_	ta
GA_4_	ta
GA_19_	ta
GA_44_	ta
GA_53_	ta
jasmonic acid (JA)	213.2 ± 48.3
salicylic acid (SA)	1830.8 ± 231.7
benzoic acid (BeA)	10,770.4 ± 426.6
abscisic acid (ABA)	ta

**Table 4:** MRM parameters at positive ion mode (+ESI), capillary voltage 4 kV, gas temperature 350 °C, gas flow 12 L/min and nebulizer pressure 35 psi. MassHunter software was used to control the LC–MS/MS system and in data analysis. For MRM parameters optimization MassHunter Optimizer was used. Monitored compounds: [^2^H_5_]benzoic acid (D-BeA), benzoic acid (BeA), [^2^H_4_]salicylic acid (D-SA), salicylic acid (SA), [^2^H_5_]jasmonic acid (D-JA), jasmonic acid (JA), [^2^H_5_]indole-3-acetic acid (D-IAA) indole-3-acetic acid (IAA), indole-3-carboxylic acid (I3CA), indole-3-acetyl-aspartic acid (IAA-Asp), gibberellic acid A_3_ (GA3), [^2^H_2_]gibberellic acid A_1_ (D-GA_1_), [^2^H_2_]gibberellic acid A_6_ (D-GA_6_), gibberellic acid A_6_ (GA6).

Compound		Type of Ion	Transition (Precursor/Product Ions)	Fragmentor Voltage (V)	Collision Energy (V)
D-BeA	ISTD	[M+H]+	128.1/84.2	60	13
BeA		[M+H]+	124.1/80.0	60	13
D-SA	ISTD	[M+H]+	143.2/125.2	80	13
			143.2/69.1		29
SA		[M+H]+	139.2/121.2	80	13
			139.2/65.1		29
D-JA	ISTD	[M+H]^+^	216.3/153.2	80	5
			216.3/135.2		9
JA		[M+H]^+^	211.3/151.2	80	14
			211.3/133.2		14
D-IAA	ISTD	[M+H]+	181.1/135.1	38	14
			181.1/82.1		53
IAA		[M+H]+	176.1/130.3	51	9
			176.1/77.2		53
I3CA		[M+H]^+^	162.2/118.1	58	9
			162.2/89.1		37
IAA-Asp		[M+H]^+^	291.2/130.1	54	13
			291.2/77.1		81
GA3		[M-H2O+H]+	329.3/283.3	100	14
			329.3/311.3		14
D-GA1	ISTD	[M-H_2_O+H]^+^	333.3/287.2	58	9
			333.3/128.1		81
D-GA6	ISTD	[M-H_2_O+H]^+^	331.3/285.3	104	9
			331.3/303.1		9
GA6		[M-H2O+H]+	329.3/283.3	104	9
			329.3/301.1		9

## Data Availability

The data presented in this study are available on request from the corresponding author.

## Supplementary Materials

The following supporting information can be downloaded at: https://www.mdpi.com/article/10.3390/ijms24032174/s1.
