# Supplementary material for: Seeds of Stevia rebaudiana Bertoni as a Source of Plant Growth-Promoting Endophytic Bacteria with the Potential to Synthesize Rebaudioside A
Source: Int J Mol Sci. 2023 Jan 21;24(3):2174. doi: 10.3390/ijms24032174 (PMC9917351; doi:10.3390/ijms24032174)

Supplementary File 1

Table S1. BLASTn homology analysis of 16S rRNA gene fragment of the SRS89 strain.

	Accession No.	Query Cover	E value	Per. Ident.
***Pantoea agglomerans* strain DSM 3493**	NR_041978	100%	0.0	99.54%
***Pantoea agglomerans* strain NCTC9381**	NR_114735	100%	0.0	99.23%
***Pantoea agglomerans* strain NBRC102470**	NR_114111	100%	0.0	99.31%
***Pantoea vagans* strain LMG24199**	NR_116115	100%	0.0	99.08%

Figure S1. Alignment of 16S rRNA gene fragment of the SRS89 strain to *P. agglomerans* (NR_041978, NR_114735, NR_114111) and *P. vagans* (NR_116115) strains.

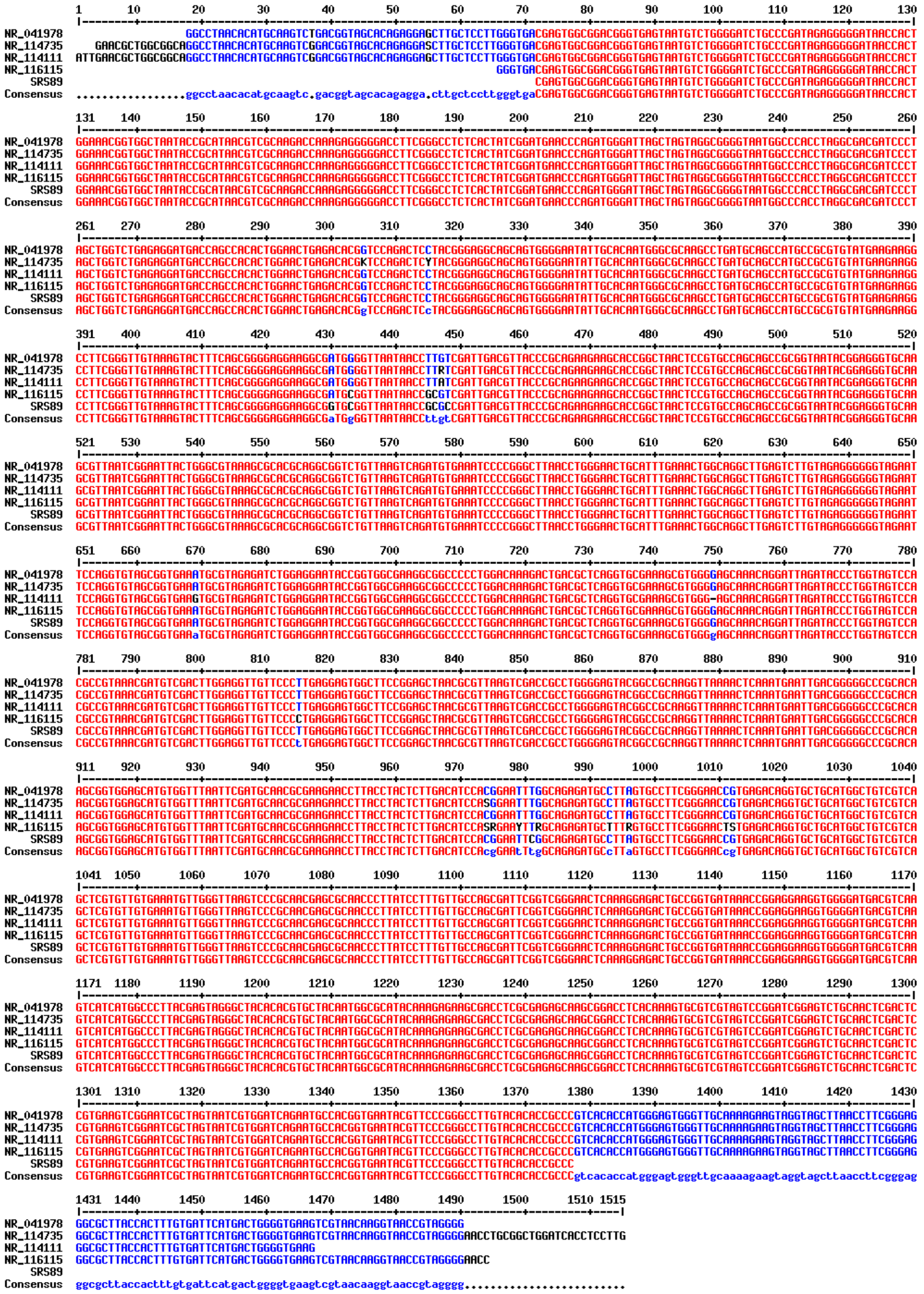

Supplement: Supplementary file 1 [file ijms-24-02174-s001.zip › Suplementary File 1_20.01.2023.pdf]
